# Design of a Single Channel Modulated Wideband Converter for Wideband Spectrum Sensing: Theory, Architecture and Hardware Implementation

**DOI:** 10.3390/s17051035

**Published:** 2017-05-04

**Authors:** Weisong Liu, Zhitao Huang, Xiang Wang, Weichao Sun

**Affiliations:** 1 College of Electronic Science and Engineering, National University of Defense Technology, Changsha 410073, China; huangzhitao@nudt.edu.cn (Z.H.); christopherwx@163.com (X.W.); 2 Southwest Electronics and Telecommunication Technology Research Institute, Chengdu 610041, China; sweichao1266@163.com

**Keywords:** cognitive radio, wideband spectrum sensing, compressed sensing, modulated wideband converter, prototype system

## Abstract

In a cognitive radio sensor network (CRSN), wideband spectrum sensing devices which aims to effectively exploit temporarily vacant spectrum intervals as soon as possible are of great importance. However, the challenge of increasingly high signal frequency and wide bandwidth requires an extremely high sampling rate which may exceed today’s best analog-to-digital converters (ADCs) front-end bandwidth. Recently, the newly proposed architecture called modulated wideband converter (MWC), is an attractive analog compressed sensing technique that can highly reduce the sampling rate. However, the MWC has high hardware complexity owing to its parallel channel structure especially when the number of signals increases. In this paper, we propose a single channel modulated wideband converter (SCMWC) scheme for spectrum sensing of band-limited wide-sense stationary (WSS) signals. With one antenna or sensor, this scheme can save not only sampling rate but also hardware complexity. We then present a new, SCMWC based, single node CR prototype System, on which the spectrum sensing algorithm was tested. Experiments on our hardware prototype show that the proposed architecture leads to successful spectrum sensing. And the total sampling rate as well as hardware size is only one channel’s consumption of MWC.

## 1. Introduction

Nowadays, with the advancement of modern communication technology, the demand for spectrum resources is growing heavily. Authorized users are suffering from heavy interference caused by other users sharing the same spectrum. Cognitive radio (CR) is regarded as a promising technology which can fully use the spectrum by dynamically accessing the currently unused licensed spectrum [1]. In this technology, obviously, spectrum sensing is a crucial task, which requires not only high reliability and short sensing time but also the ability to sense a wide bandwidth. Customarily, there are three common spectrum sensing techniques [2,3,4]. The first approach is called matched filter which requires a prior knowledge of the primary users signaling features such as bandwidth, operating frequency, modulation type, pulse shaping and so on. However, these are usually infeasible to acquire in practice. The second is energy detector, it is the most common way of spectrum sensing owing to its low computation and implementation complexities. However, its sensing accuracy is highly susceptible to noise uncertainty and becomes unacceptable when the signal-to-noise-ratio (SNR) is pretty low. The third one is cyclostationary feature detector. Although this way is quite robust to both noise uncertainty and strong noise, it still takes a long time due to high computational complexities. Besides, all the approaches mentioned above apply to the narrow band signals or low frequency field.

When it comes to wideband spectrum, in [5], two possible ways are discussed. One is to divide the whole wideband spectrum into lots of narrow band channels and then we can carry out the above sensing approaches channel-by-channel. Similarly, this approach introduces a big time delay and a significant amount of bandpass filters because of its serial process. Another approach is to directly sample the entire wideband spectrum using a high-rate analog-to-digital converter (ADC). However, under Nyquist sampling framework, such high sampling rate may exceed today’s best ADCs performance. What is more, this will generate a large number of samples to process, affecting speed and power consumption.

Many studies have been performed to overcome this bottleneck. In [6], Landau developed a minimal sampling rate requirement that allows perfect reconstruction as early as 1967. Unfortunately, the article did not point out a feasible method of implementation when the carrier frequencies are unknown. The recent theory of compressed sensing (CS) [7,8], indicates that if the signal has a sparse representation in some space, we can linearly transform the signal to a lower-dimension space from which the signals can be recovered without any distortion. In this strategy, the sampling rate is no longer determined by the highest frequency of the signal, but decided by its valid information. Based on this, a number of CS structures have been proposed for sub-Nyquist sampling of continuous-time signals in recent years. Such as random demodulation (RD), Multi-coset sampling, modulated wideband converter (MWC) and so on [9,10,11,12]. However, the RD system can only treat the multi-tone signal model. In reality, the analog signals require a extremely large number of harmonics to approximate them well within the discrete model, which may cause the reconstruction computationally infeasible. Multi-coset sampling system has a good performance of the multiband signal. However, this scheme is fairly hard to implement because maintaining accurate time shifts, on the order of the Nyquist interval, is extremely difficult to design and realize. Lately, Mishali et al. proposed MWC sampling structure. Through a series of processing steps on a special analog front-end, the signal can be reconstructed from low rate samples, using the known relation between samples and the original signal. It is the most striking structure and can be realized by commercial components. Whereas, the amount of sampling channels can become very large when the sparsity is great [13]. Its high hardware complexity is unacceptable for a single node the in the application of CR. Besides, the synchronization of each parallel channel is difficult to guarantee.

However, considering our ultimate goal is spectrum sensing and detection, there is no need to reconstruct the original signal. Consistent with the idea presented in [5,14], we plan to sense the power spectrum of the interested bandwidth from sub-Nyquist samples. Meanwhile, in order to simplify the hardware complexity, we intend to explore a more succinct architecture. In our previous work [15], we first proposed a single channel modulated wideband converter for power spectrum sensing and reconstruction with some expressions and simulations. However, in order to apply in CR system, parament selections, prototype design as well as algorithm modification need further research.

In this paper, a new spectrum sensing hardware prototype based on single channel modulated wideband converter (SCMWC) was designed and tested by actual circuits. It has the ability to treat analog multiband models as well as MWC. More importantly, practical implementation, low hardware complexity and low computational load make it possible to be used for CR system. Our main contribution of this paper is in proving feasibility of the SCMWC strategy using commercial devices. In detail, it can be divided into two parts. The first one is that we design and implement a hardware prototype based on SCMWC scheme. The proposed SCMWC hardware prototype only consists of one modulator, low-pass filter (LPF) and low rate ADC. Particularly, we introduce Rocket I/O on FPGA to generate high speed periodic sequences. By using this, the implementing process is simplified. The second one is that a series of simulations and experiments are conducted to acquire the proper parameters as well as evaluate the performance of the hardware prototype. Results show that our design can treat 2 GHz Nyquist-rate input signals with spectrum occupancy up to 15 MHz. And the total equivalent sampling rate is only 70 MHz, which is only 3.5% of the Nyquist rate. Besides, the high dynamic range and low complexity make it more suitable for being applied to CR system.

The rest of this paper is organized as follows. Section 2 describes the spectrum sensing model. Then the architecture of SCMWC is presented and analyzed in Section 3. This leads to a parameter selection which guarantees successful sensing from the digital samples. Section 4 detailed describes the design methods and exact specs of the proposed circuit board. In Section 5, we present the some simulations and experiments based on our prototype. Besides, comparisons with related work are discussed. Finally, Section 6 concludes the paper.

## 2. Spectrum Sensing Model

Now we consider a CR system which is in a hot-spot area, several studies [14,16] have shown that across a wide spectrum range, the spectrum can be expressed as a union of some narrow-band transmissions. Our main ambition is to find out the signal support, which means the bands of the signal sources reside. In other words, we intend to sense the spectrum of the current situation at sub-Nyquist rates.

Suppose that the received signal from one spectrum sensor x(t) consists of $N_{x}$ uncorrelated stationary transmissions, which can be formulated like this

(1) $$x\left(t\right)=\sum_{i=1}^{N_{x}}\rho_{i}s_{i}\left(t\right).$$

Here, $s_{i}\left(t\right)$ is a zero-mean bandlimited wide-sense stationary (WSS) signal, and $\rho_{i}\in \left\{0,1\right\}$ indicates that whether the *i*th signal is active or not.

**Figure 1:**
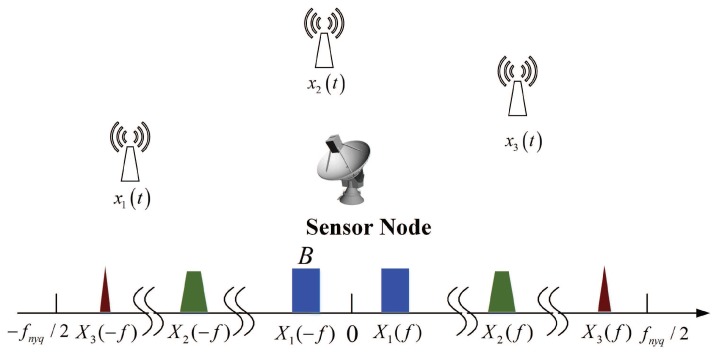
Typical spectrum support of a multiband signal composed of three RF transmissions.

Figure 1 depicts a typical single node spectrum sensing model and spectrum support of a multiband signal. There are three RF transmissions with different carriers $f_{i}$ over the whole spectrum. The Fourier transform of x(t) is expressed by X(f). $f_{nyq}$ denotes the Nyquist rate of x(t) and $S_{x}$ is the support of the Fourier transform X(f). As is assumed in [11], we consider the same scenario: we have no prior knowledge on the carrier frequencies of the spectrally sparse continuous-time signal x(t) of finite support on $F_{nyq}=\left[-f_{nyq}/2,+f_{nyq}/2\right]$, and we suppose that the maximal number of signals is $N_{x}$ with maximal bandwidth *B*. We can see that the support $S_{x}$ is contained within a union of $N=2N_{x}$ disjoint bands in $F_{nyq}$.

It is well known that the power spectrum of x(t) is given by

(2) $$P_{x}\left(f\right)=\int_{-\infty }^{\infty }r_{x}\left(\tau \right)e^{-j2\pi f\tau }d\tau ,\;f\in F_{nyq}$$

where $r_{x}\left(\tau \right)=E\left[x\left(t\right)x\left(t-\tau \right)\right]$ represents the autocorrelation function of x(t) and E[·] denotes the expectation operation. From Equation (2), we can see that estimating the power spectrum equals to estimating the autocorrelation function $r_{x}\left(\tau \right)$. Obviously, $P_{x}\left(f\right)$ has the same support with X(f). So sensing the spectrum changes into estimating the power spectrum.

## 3. Single Channel Modulated Wideband converter

### 3.1. Structure Description

**Figure 2:**
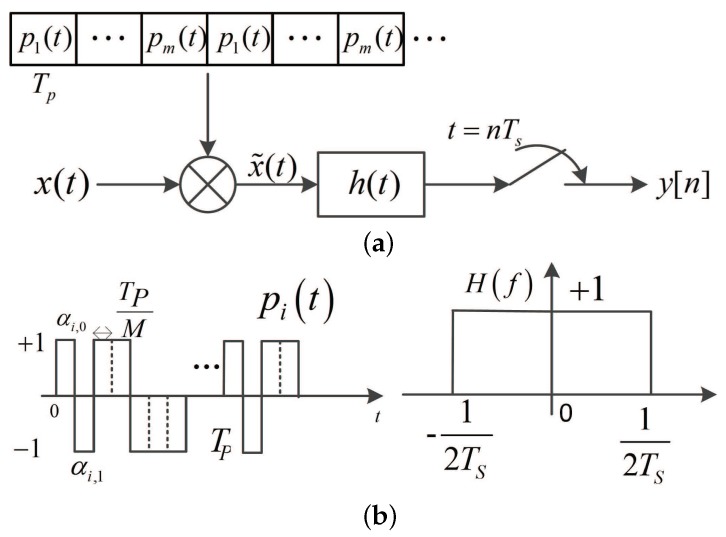
The Schematic Diagram of a single channel modulated wideband converter (SCMWC) sampling scheme (**a**). Periodic Waveform and low-pass filter (LPF) Frequency Response (**b**).

The structure of SCMWC is shown in Figure 2. It consists of a mixer, a low-pass filter and a low-rate ADC. As shown in **a**, the input signal x(t) is firstly multiplied by the mixing periodic sequences $p_{i}\left(t\right)$, which is described in **b** (left drawing). Then it is filtered by the low-pass filter h(t) with cutoff frequency $1/2T_{s}$ shown in **b** (right drawing). Finally the output signal is sampled at rate $f_{s}=1/T_{s}$. The $p_{i}\left(t\right)$ is a piecewise constant function that alternates between the levels ±1 for each of *M* equal time intervals. Its period is $mT_{p}$ and there are *m* different sequences with the same time length $T_{p}\left(1/T_{p}\geq B\right)$, which have no correlation with each other. Now we define $T_{i}=\left[\left(i-1\right)T_{p}+nmT_{p},iT_{p}+nmT_{p}\right]$ (n∈N), and it is obvious that the $p_{i}\left(t\right)$ in the interval $T_{i}$ is $T_{p}$-periodic.

### 3.2. Spectrum Analysis

At first, we introduce a definition $g_{T}\left(t\right)=\left\{\begin{array}{c} 1,\;|t|\leq T/2\\ 0,\;|t|>T/2 \end{array}\right.$. Then, the input signal x(t) in $T_{i}$ can be expressed as

(3) $$x_{i}\left(t\right)=x\left(t\right)g_{T_{p}}\left(t-t_{i}\right),\;t_{i}=\left(i-1\right)T_{p}+T_{p}/2+nmT_{p}$$

**Figure 3:**
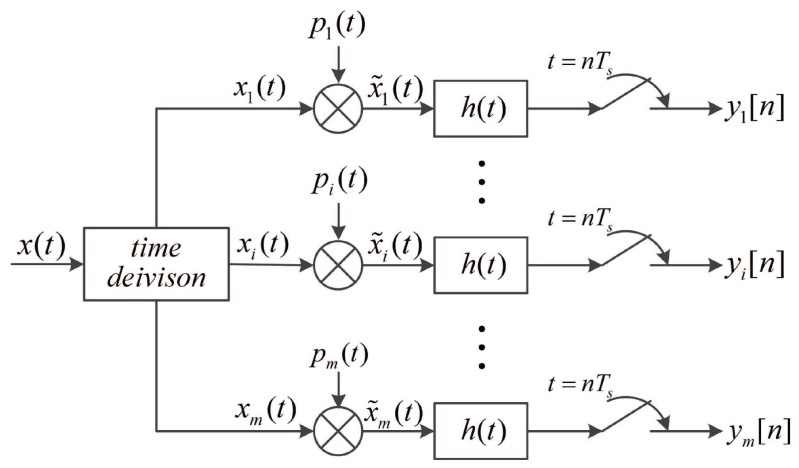
The equivalent of proposed single channel sub-Nyquist sampling scheme.

And the random sequence $p_{i}\left(t\right)$ in $T_{i}$

(4) $$p_{i}\left(t\right)=a_{ik},\;kT_{p}/M\leq t\leq \left(k+1\right)T_{p}/M$$

with $a_{ik}\left\{+1,-1\right\}$, 1≤i≤m, 0≤k≤M−1. And *M* is a prime number representing the number of intervals in each $p_{i}\left(t\right)$. For convenience of explanation, we present a equivalent structure in Figure 3.

Since $p_{i}\left(t\right)$ is a periodic function, the Fourier expansion can be expressed as

(5) $$p_{i}\left(t\right)=\sum_{l=-\infty }^{\infty }c_{il}e^{j2\pi lf_{p}t}$$

where $c_{il}=\frac{1}{T_{p}}\int_{0}^{T_{p}}p_{i}\left(t\right)e^{-j2\pi lf_{p}t}dt$. Then, after mixing, the Fourier transform of the *i*th output signal ${\tilde{x}}_{i}\left(t\right)=x_{i}\left(t\right)p_{i}\left(t\right)$ is

(6) $${\tilde{X}}_{i}\left(f\right)=\int_{-\infty }^{\infty }x_{i}\left(t\right)\left(\sum_{l=-\infty }^{\infty }c_{il}e^{j2\pi lf_{p}t}\right)e^{-j2\pi lft}dt=\sum_{l=-\infty }^{\infty }c_{il}X_{i}\left(f-lf_{p}\right)$$

where $X_{i}\left(f\right)$ is the Fourier transform of the *i*th time interval of x(t). We can see that before LPF filtering process, it is a linear combination of $f_{p}$-shifted copies of $X_{i}\left(f\right)$.

Due to the low pass filter, only frequencies in the interval $F_{s}=\left[-f_{s}/2,f_{s}/2\right]$ can appear in the final output sequence $y_{i}\left[n\right]$. So the discrete-time Fourier transform (DTFT) of $y_{i}\left[n\right]$ is

(7) $$Y_{i}\left(e^{j2\pi fT_{s}}\right)=\sum_{l=-\infty }^{\infty }y_{i}\left[n\right]e^{-j2\pi fnT_{s}}=\sum_{l=-L_{0}}^{L_{0}}c_{il}X_{i}\left(f-lf_{p}\right),\;f\in F_{s}$$

where $L_{0}=\left\lceil \frac{f_{nyq}+f_{s}}{2f_{p}}\right\rceil -1$, and let $L=2L_{0}+1$, which represents the number of spectrum bands we divided.

Now, we can write Equation (7) in matrix form as

(8) $$y\left(f\right)=Az\left(f\right),\;f\in F_{s}\;,$$

with $y\left(f\right)=diag\left\{Y_{i}\left(e^{j2\pi fT_{s}}\right)\right\}$, $A=diag\left\{C_{i}\right\}$, $C_{i}=\left[\begin{array}{cc} c_{i,-L_{0}} & c_{i,-L_{0}+1} \end{array}\begin{array}{cc} \cdots & c_{i,L_{0}} \end{array}\right]$, $z\left(f\right)=diag\left\{z_{i}\right\}$, $z_{i}\left(f\right)={\left[X_{i}\left(f+L_{0}f_{p}\right)\;X_{i}\left(f+\left(L_{0}-1\right)f_{p}\right)\cdots X_{i}\left(f-L_{0}f_{p}\right)\right]}^{T}$, i=1,…m, and ${\left(\cdot \right)}^{T}$ denotes transpose operation.

### 3.3. Power Spectrum Sensing

We aim to derive the relation between the sample sequences $y\left[n\right]$ and the unknown power spectrum $P_{x}\left(f\right)$. Since x(t) is a wide-sense stationary process, in $T_{i}$, the relation between $X_{i}\left(f\right)$ and y(f) can be expressed as follows

(9) $$E\left[{\left|X_{i}\left(f\right)\right|}^{2}\right]=E\left[{\left|X(f)\right|}^{2}\right]=P_{x}\left(f\right),\;1\leq i\leq m$$

where $E\left[\cdot \right]$ denotes the expectation operator. $X_{i}\left(f\right)$ is the Fourier transform of $x_{i}\left(t\right)$, and $P_{x}\left(f\right)$ is given by Equation (2). Then we have

(10) $$E\left[X_{i}\left(f_{1}\right)X_{i}^{*}\left(f_{2}\right)\right]=P_{x}\left(f_{1}\right)\delta \left(f_{1}-f_{2}\right),\;1\leq i\leq m$$

where $f_{1}$ and $f_{2}$ are any frequencies within $f_{nyq}$.

We define the autocorrelation matrix

(11) $$R_{z}\left(f\right)=E\left[z\left(f\right)z^{H}\left(f\right)\right]\;,$$

where ${\left(\cdot \right)}^{H}$ denotes the Hermitian operation. From Equation (10), we know that $R_{z}\left(f\right)$ is a block diagonal matrix with block diagonal element $R_{z}^{i}\left(f\right)=E\left[z_{i}\left(f\right)z_{i}^{H}\left(f\right)\right]$, $R_{z}^{i}\left(f\right)$ is also a diagonal matrix with diagonal line element $R_{z}^{i}\left(j,j,f\right)=P_{x}\left(f+l_{j}f_{s}\right)$, where $l_{j}=-L_{0}+j-1$, 1≤j≤L. Obviously, our aim can be described as recovery of $R_{z}\left(f\right)$, because once $R_{z}\left(f\right)$ is known, $P_{x}\left(f\right)$ follows for all *f*.

Now, we relate $R_{z}\left(f\right)$ to the correlation of the sub-Nyquist samples. From Equation (8), we have the relationship in matrix form as

(12) $$R_{y}\left(f\right)=AR_{z}\left(f\right)A^{H},\;f\in F_{s}\;$$

Besides, $R_{y}\left(f\right)$ can be expressed

(13) $$R_{y}\left(f\right)=E\left[y\left(f\right)y^{H}\left(f\right)\right]=\left[\begin{array}{ccc} E[Y_{1}\left(e^{j2\pi fT_{s}}\right)Y_{1}^{H}\left(e^{j2\pi fT_{s}}\right)] & & 0\\ & \ddots & \\ 0 & & E[Y_{m}\left(e^{j2\pi fT_{s}}\right)Y_{m}^{H}\left(e^{j2\pi fT_{s}}\right)] \end{array}\right]\;$$

where the diagonal line element of $R_{y}\left(f\right)$ is

(14) $$R_{y}\left(i,i,f\right)=E\left[Y_{i}\left(e^{j2\pi fT_{s}}\right)Y_{i}^{H}\left(e^{j2\pi fT_{s}}\right)\right]=C_{i}R_{z}^{i}\left(f\right)C_{i}^{H}=\sum_{l_{i}=-L_{0}}^{L_{0}}|c_{i,l_{i}}{|}^{2}P_{x}\left(f-l_{i}f_{s}\right),\;f\in F_{s}\;$$

For ease of description, we define

(15) $$r_{y}\left(f\right)={\left[R_{y}\left(1,1,f\right)\;R_{y}\left(2,2,f\right),\cdots ,R_{y}\left(m,m,f\right)\right]}^{T},\;f\in F_{s}\;$$

(16) $$\Phi =A.*\bar{A}$$

and

(17) $$r_{x}\left(f\right)={\left[P_{x}\left(f+L_{0}f_{s}\right)\;P_{x}\left(f+\left(L_{0}-1\right)f_{s}\right),\cdots ,P_{x}\left(f-L_{0}f_{s}\right)\right]}^{T}$$

where (.*) denotes pointwise matrix multiplication and $\left(\bar{\cdot }\right)$ denotes complex conjugacy. From Equation (14), finally we can get the matrix equation between the power spectrum $P_{x}\left(f\right)$ and samples as follows

(18) $$r_{y}\left(f\right)=\Phi \;r_{x}\left(f\right),\;f\in F_{s}\;$$

Then we can recover the entire power spectrum of x(t) by recovering $r_{x}\left(f\right)$ for all $f\in F_{s}$.

Because of the larger of *m*, the longer of sensing time needed. In general, the Equation (18) is a underdetermined function (m<L). So, next we should solve the undetermined function to detect the support.

For this purpose, we adopt a operation called continuous-to-finite (CTF) detailed in [11]. From Equation (18), we have

(19) $$Q=\Phi \;Z\;\Phi^{H},\;f\in F_{s}$$

where $Q=\int_{f\in F_{s}}r_{y}\left(f\right)r_{y}^{H}\left(f\right)df$ and $Z=\int_{f\in F_{s}}r_{x}\left(f\right)r_{x}^{H}\left(f\right)df$. Then, any matrix V, for which $Q=VV^{H}$, is a frame for $r_{y}\left(F_{s}\right)$ [17]. We construct it by performing an eigendecomposition of Q and choosing V as the matrix of eigenvectors corresponding to the non-zeros eigenvalues. Through solving the linear equations V=ΦU for a unique sparsest solution, we will get the the support which is the same as the support $S_{x}$ of Equation (18). In compressed sensing theory, there are many ways to solve this, such as Basis pursuit [18] and Matching pursuit [19] and so on. Here we use a relatively faster algorithm Orthogonal Match Pursuit (OMP). So far, through the analysis above, we have shown the way to detect the support $S_{x}$.

## 4. Circuit Description

**Figure 4:**
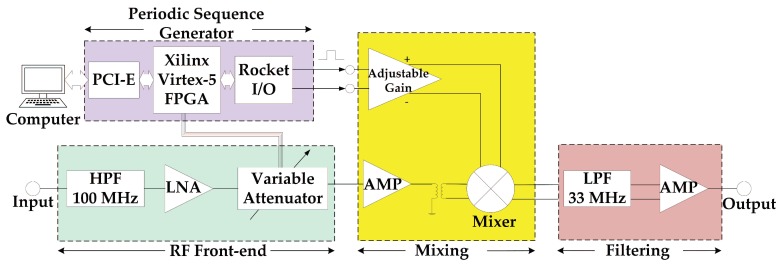
Block diagram of the proposed circuit board based on SCMWC.

In this section, we present a printed circuit board (PCB) which is designed to demonstrate the ability of the SCMWC for spectrum sensing. The board consists of four parts: RF front-end, periodic sequence generator, mixer and low pass filter. The input signal is firstly fed to the RF front-end so as to catch the interested signal. Then the output signal is mixed with a periodic waveform through a wideband active mixer. Finally the output of the mixer is low-pass filtered before being sampled. In our lab experiments, we use a oscilloscope to sample and store the waveform for simplification. However, in the future, we will use commercial ADC devices for that task. Figure 4 presents a block diagram of the board. We then describe each part in detail.

### 4.1. Hardware Specifications

From the sampling theory above, we can see that the SCMWC is a flexible system with several parameters. Table 1 shows the specifications we choose for circuit level implementation.

To begin with, we should specify the multiband signal model, N=4, B=15 MHz, $f_{nyq}=2$ GHz. That is to say we can sense two bands at the same time and each band is no more than 15 MHz. The alternation rate of the periodic sign is 2 GHz, which is generated by Gigabit Transceiver X (GTX) on Xilinx FPGA. Based on the theory in [11], the basic parameter choice is m≥4N and $f_{s}=f_{p}\geq B$. In order to reduce sensing time, we choose the minimum number m=4N=16. For convenience, we set the cut-off frequency of LPF at 33 MHz, owing to the difficulty in implementing a much lower cut-off frequency.

### 4.2. Circuit Module Design

#### 4.2.1. RF Front-End

**Figure 5:**
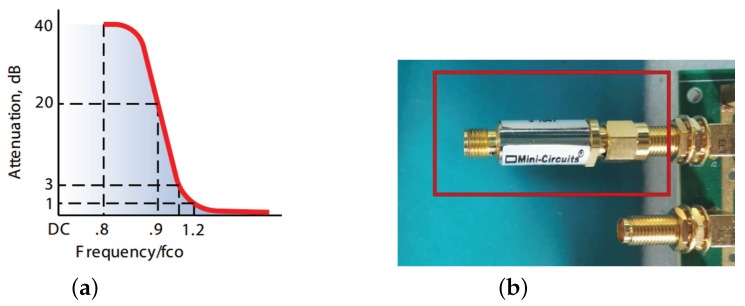
The frequency response (**a**) and a photograph (**b**) of the high pass filter (HPF).

The analog RF front-end consists of a high pass filter (HPF), one low noise amplifier (LNA) and a variable attenuator. With the HPF as off-board components, the system stays highly flexible. We choose SHP-100A+ as HPF to remove some low-frequency noise, which can just meet thee demand of specifications in Table 1. Figure 5 depicts its actual shape and typical frequency response.

**Figure 6:**
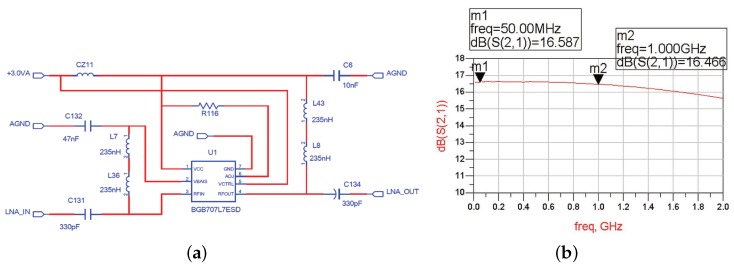
The circuit diagram (**a**) and frequency characteristic (**b**) of low noise amplifier (LNA).

The signal from the HPF is then fed to the input of LNA. LNA is one of the essential building blocks of RF receivers. We choose a high performance broadband LNA MMIC BGB741L7ESD from Infineon to be the first stage amplifier. The noise figure is lower than 2 dB and the gain is more than 16 dB. Before designing the circuit, we make several simulations through the Advanced Design System (ADS) to select the appropriate parameters. The final circuit and frequency characteristic are shown in Figure 6. We can see that the amplifier gain is stable around 16.5 dB, and the passband fluctuation is less than 3 dB. In addition, if the amplitude of input signal is very high, the LNA can be bypassed and directly fed to the variable attenuator.

**Figure 7:**
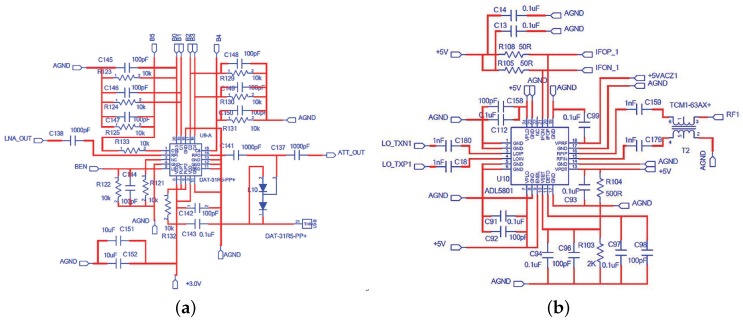
The circuit diagram of attenuator (**a**) and modulator (**b**).

In order to increase the dynamic range, we use a digital step attenuator DAT-31R5A-PP+ from Mini-Circuits to prevent saturation of the back-end devices. It provides adjustable attenuation from 0 to 31.5 dB in 0.5 dB steps. The control is a 6-bit parallel interface, operating on single supply voltage. We connect the control logic interface and FPGA logic I/O. Then the desired attenuation state can be selected through man-machine interactive system. The schematic of the RF attenuator is attached in Figure 7a.

#### 4.2.2. Mixing Module

In [20], they encountered two main difficulties when designing an analog circuit to realize the MWC. One is analog mixing with spectrally rich waveforms, and another is constructing the periodic waveforms with a required alternation speed. Similarly in SCMWC, to solve the first problem, one of the essential blocks is the mixer. Since $p_{i}\left(t\right)$ spans a wide spectrum, the mixer should allow a wide range of LO frequencies. We choose the wideband active mixer chip ADL5801 produced by Analog Device. This kind of mixer uses a high linearity, doubly balanced, active mixer core with integrated LO buffer amplifier to provide high dynamic range frequency conversion from 10 MHz to 6 GHz [21]. The LO circuit exhibits low additive noise, resulting in an excellent mixer noise figure. As for RF, for the sake of optimal performance, a balun is chosen to convert the single-ended signal into two differential signals. The schematic of the modulator is shown in Figure 7b.

#### 4.2.3. Periodic Sequence Generator

In order to solve the problem of generating high rate periodic sequences, some approaches have been discussed in [20]. Finally, a special way is adopted. Several shift-registers are connected in series becoming a shift-register circle. Before the register works, the initial value of each register has to be set by switches. Then four channels of sequences come from four different taps. However, in this way the sequences have high correlation, which may leads to reconstruction failure. Fortunately, we learn that the Xilinx FPGA chip is equipped with Rocket I/O transceiver. And the GTZ (Gigabit Transceiver Z) transceivers are capable of running up to 28.05 Gb/s [22]. Each transceiver includes an independent transmitter and receiver. Based on this, we come up with an idea to use a transceiver to transmit the sequences without receiving them. Finally, we choose the chip Virtex-5 series XC5VFX30T with four GTX transceivers as the main controller. We then use one GTX transmitter to generate periodic sequences. The sequences are written in a ROM firstly. Then the transceiver is controlled to output the sequences circularly. As for the interface between the mixer and transmitter, since the output of the transmitter is Current Mode Logic (CML), it is just compatible with the LO of the mixer [23].

**Figure 8:**
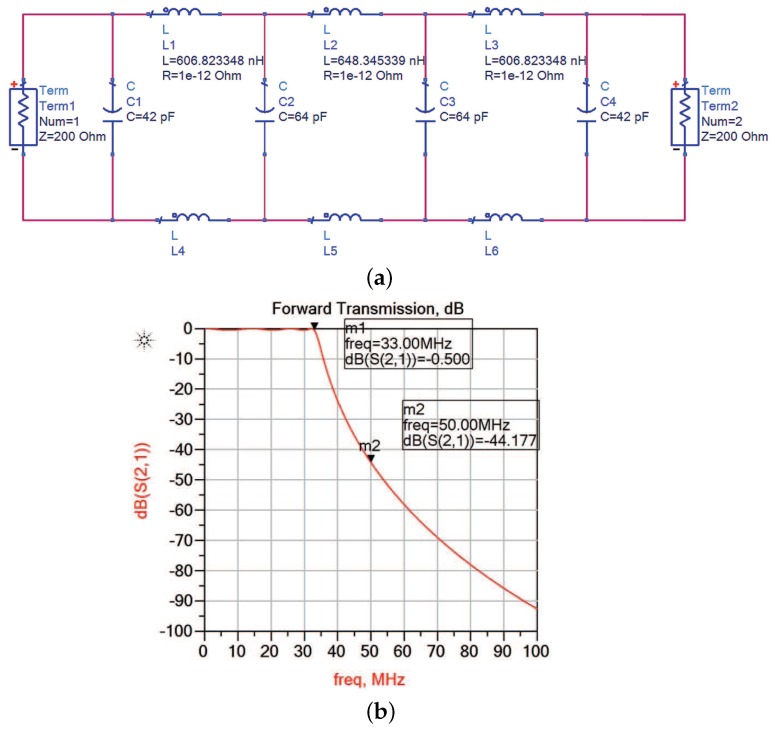
The circuit diagram (**a**) and frequency characteristic (**b**) of low-pass filter (LPF).

From Equation (6), we can see that the output of mixer contains energy spread all across the spectrum. A LPF is needed before sampling process by low rate ADC. The main purpose of the LPF is anti-aliasing filtering in case output signal exceeds the bandwidth of ADC. Besides, the filter needs to be differential corresponding with the output of mixer. We use the common simulation software Advanced Design System (ADS) to simulate a Chebyshev low pass filter of order seven. The cut-off frequency of passive filter is around 33 MHz, its circuit diagram **a** and frequency characteristic **b** are shown in Figure 8.

#### 4.2.4. LPF Module

**Figure 9:**
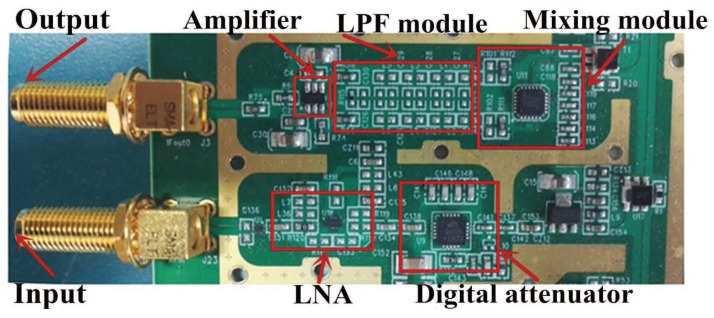
Photograph of the circuit board.

In addition, a differential amplifier OPA847 is adopted to convert differential signal into a amplifying single-end signal for the convenience of sampling process. A photo of the circuit board is attached in Figure 9.

## 5. Experiments and Discussions

**Figure 10:**
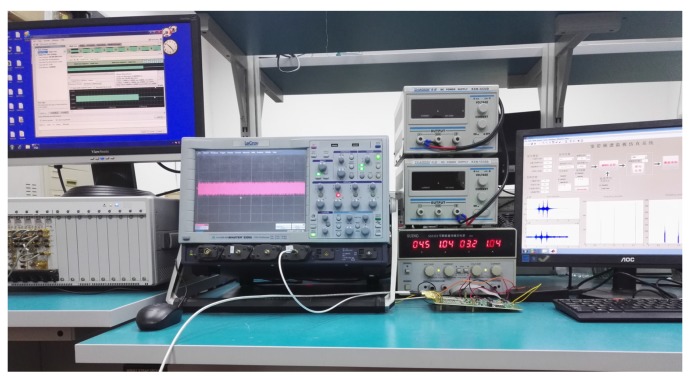
Photograph of the experiment environment.

In this section, we demonstrate the SCMWC sensing strategy using our designed system. We first explain the data-processing software, and then describe the software simulations for the system. Finally some hardware experiments we conducted to prove the performance of our circuit board. Throughout the experiments, we use several lab equipments, that include: Arbitrary Wave Generator (AWG)-Keysight M8190A (Keysight Technologies, Santa Rosa, FL, USA), Agilent N9936A (Keysight Technologies), LeCroy 8500A oscilloscope (Teledyne LeCroy, Chestnut Ridge, NY, USA) and a desktop computer, etc. Figure 10 shows the main elements in our experiment setup.

### 5.1. Background: Software Design

**Figure 11:**
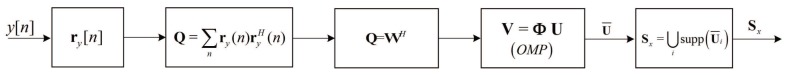
Block diagram of spectrum sensing algorithm.

To get the samples, for convenience, we used the above oscilloscope instead of commercial ADCs. Firstly, according to the Nyquist sampling theory, we set the sampling rate at 100 MHz, which is the minimal appropriate sampling rate of the oscilloscope. As soon as the waveform is stored, it is transfered to the back-end computer for software processing. In designing the software, we divided the whole process into several steps as follows:

1. Calibration. This step is essential, because the relation between the samples and the original signal becomes unknown in real hardware implementation. Physical effects have a considerable impact on the sampling process, which may lead to detection failure [24]. However in [25], an automated calibration algorithm is presented applying to MWC. Similarly, in SCMWC, same process is adopted and implemented in our software. Generally, once the circuit board is fixed, the relation becomes a constant matrix. So the calibration process only need once.
2. Digital Finite Impulse Response filtering. Actually, the SCMWC requires ideal analog low pass filters to accomplish the detection process. however, in practice, implementing ideal filters is generally difficult. Usually, people use digital filters to compensate for the analog filters. It is proved in [26] that with only a moderate amount of oversampling, the imperfections caused by non-ideal filters can be effectively corrected in the digital domain. In our software, the samples from the oscilloscope are firstly put through a digital Finite Impulse Response (FIR) filter with cutoff frequency 7.75 MHz ($1/2f_{p}$). Then, we resample the output data, to ensure the actual sampling rate is a little higher than $f_{p}$. At this point, the preprocessing of the data is complete.
3. Power spectrum sensing. Every piece of the data is then processed automatically by the software. As described in Section 3.3, we divide the sensing algorithm into several steps shown in Figure 11. Firstly, we get the autocorrelation matrix $r_{y}\left(n\right)$ from samples y(n). The frame Q is then calculated. Next we perform eigenvalue decomposition to get V which has the same column space as $r_{y}\left(n\right)$, and the rank of the two matrixes are the same. Finally, we use some common CS algorithms to solve the frequency support. When the support $S_{x}$ is known, the signal frequency position is certain.
  (20) $${\hat{r}}_{x}^{S_{x}}\left(f\right)=\Phi_{S_{x}}^{†}r_{y}\left(f\right),\;f\in F_{s}\;,$$
  where submatrix $\Phi_{S_{x}}$ contains the columns of Φ indexed by $S_{x}$ and the $\Phi_{S_{x}}^{†}={\left(\Phi_{S_{x}}^{H}\Phi_{S_{x}}\right)}^{-1}\Phi_{S_{x}}^{H}$ is the Moore-Penrose pseudoinverse of $\Phi_{S_{x}}$

### 5.2. Software Simulations

**Figure 12:**
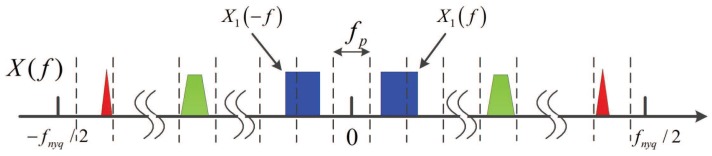
Schematic of a signal occupying two bands.

Before simulations, we have to define the successful detection as ${\hat{S}}_{x}⊇S_{x}$ rather than ${\hat{S}}_{x}=S_{x}$, where ${\hat{S}}_{x}$ is the the detected support. Because that if the band-limited signal occupies two bands like $X_{1}\left(f\right)$ as shown in Figure 12, this signal will have two neighboring supports after calculating. In general, before the CS algorithms solving the Equation (18), we need to know the largest possible sparsity. In order to avoid missing alarm, we set the sparsity two times of *N*. So estimating the success detection equals to deciding whether $S_{x}$ is included in ${\hat{S}}_{x}$.

However, acquiring the solution $S_{x}$ is NP-hard. Using some known CS algorithms can not promise 100% correct solution. However, the detection performance can be improved by increasing the sensing time. A straightforward way is to increase the parameter *m*. From [11], for practical applications, the value of *m* is roughly

(21) m≈4Nlog(M/2N)

**Figure 13:**
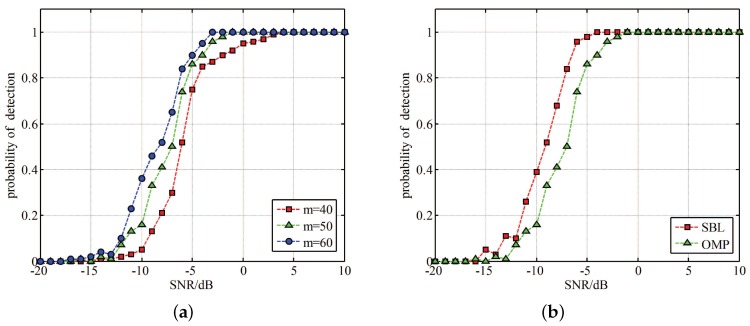
Two simulation results of the hardware prototype. (**a**) Detection performance with different *m* and SNR; (**b**) Detection performance comparison between SBL and OMP.

To evaluate the performance of the specifications we choose in Table 1 for the proposed prototype, we did some simulations on Matlab. The bandwidth we plan to sense is 1 GHz, and it is so wide that we can not generate a such wideband noise for hardware experiment. The signal to noise ratio is defined as 10log(x(t)2x2w2w(t)2), where x(t) is the wideband signal and w(t) is the wideband additive white Gaussian noise. We conducted two simulations, and each experiment is repeated over 500 realizations. The first one is to find the relationship between detection performance and SNR. As shown in Figure 13a, we choose three different empirical values of *m* according to Equation (21). We can see that the detection performance becomes better with SNR increasing. When the SNR is up to −5 dB, the successful detection rate is more than 80%. Besides, we can also find the detection probability can improve a lot with *m* increasing when the SNR is between −10 dB and −5 dB. In other SNR intervals, this effect is not significant.

As for another simulation, we introduced to use Sparse Bayesian Learning (SBL) [27] owning to its adaptation to number of signals. SBL-based method takes advantage of the probability density distribution of the data to achieve a best matching between the calculational results and the samples, which means that SBL techniques do not depend on the information maximum number of signals. This property of SBL-based techniques is suitable in our situation. we intend to apply SBL algorithm to solve the problem in SCMWC. Figure 13b depicts the probability of detection between OMP and SBL algorithm in different SNR. We set m=50 in this situation. We can see that SBL-based method has better performance, it can increase around 20% between−10 dB and −5 dB. However, SBL algorithm takes up more time than OMP, which is not appropriate in real time detection. Above all, these two simulations proves the feasibility of our prototype, and indicate the performance of the proposed specifications.

### 5.3. Hardware Experiments and Analyses

In this section, we describe several experiments based on our circuit board. The first one is showing the waveform of the period sequences generator. Then some typical parameters are analysed. At last, several spectrum sensing experiments are conducted and analysed.

#### 5.3.1. Periodic Sign Waveforms

**Figure 14:**
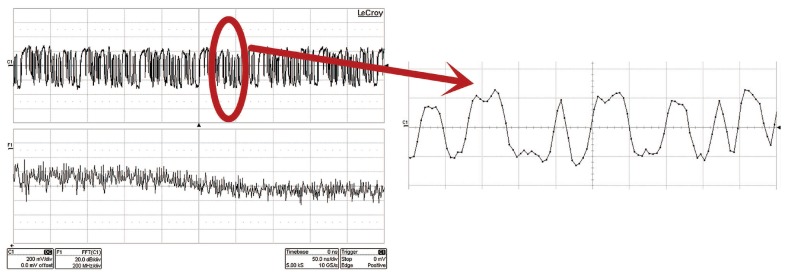
Time and frequency domain of periodic sequences.

This experiment mainly focuses on checking the performance of periodic sequences generated by GTX. Figure 14 shows the time-domain appearance and the spectrum of $p_{i}\left(t\right)$. From the time-domain, we can see the waveform is not actually square. However, its spectrum consists of concentrated energy peaks which we call Dirac functions. Corresponding to the Equation (6), we can see that the role of the periodic sequences is to move all the signal residing in different frequency bands to baseband. And this make it possible for our system to sample at a low rate and then sense it.

#### 5.3.2. Dynamic Range

As a spectrum sensor, some basic technical indicators have to be calculated, such as dynamic range and noise figure, etc.

**Figure 15:**
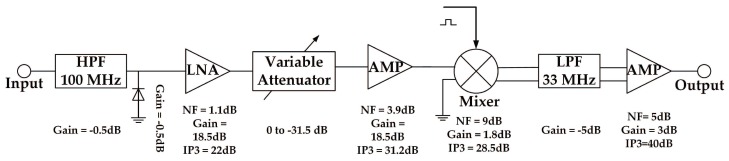
Link budget of the hardware system.

Figure 15 depicts the link budget calculation of the hardware system. To satisfy the sampling interface of oscilloscope, we set the minimal output amplitude level at 100 millivolts peak-to-peak (mVptp). It is easily to be formulated as

(22) $$P_{out}\geq -16\;\mathrm{dBm}.$$

From the simulation results in Figure 13, the SNR is at least 0dB that can ensure 100% correct detection. So

(23) SNDR≥0dB

which means the required signal to noise-and-distortion ratio. Then the minimal input power can be expressed as

(24) $$P_{in,\min}\geq P_{out}-\sum G_{i}=-16-35.8=-51.8\;\mathrm{dBm},$$

where $G_{i}$ represents the gain of each device, and the attenuator is set to 0 dB.

As for maximal input, the variable attenuator is set to the maximal level of −31.5 dB as well with the purpose of reducing enormous gain of the frontend. In order to acquire the maximal input power, we have to calculate noise figure and IP3 of the device chain first. The noise figure is formulated as

(25) $$NF=NF_{1}+\frac{NF_{2}-1}{G_{1}}+\frac{NF_{3}-1}{G_{1}G_{2}}+\ldots$$

After calculation, the equivalent NF of the system is around 3.97 dB. And the equivalent IP3 of the entire system is counted by [28]

(26) $${\left(IP3\right)}^{-1}=\sum_{i=1}^{I}{\left(IP3_{i}\prod_{j=i+1}^{I}G_{j}\right)}^{-1}={\left(27.1\;\mathrm{dB}\right)}^{-1}$$

According to [20], the

(27) $$P_{in,\max}\leq IP3-\frac{1}{2}\left(SNDR+7.3\right)-10\log_{10}N_{\max}-\sum G_{i}=9.15\;\mathrm{dBm}$$

From Equation (24) and (27), the dynamic range of our system is finally derived as 60.95 dB.

#### 5.3.3. Sensing Experiments and Results

**Figure 16:**
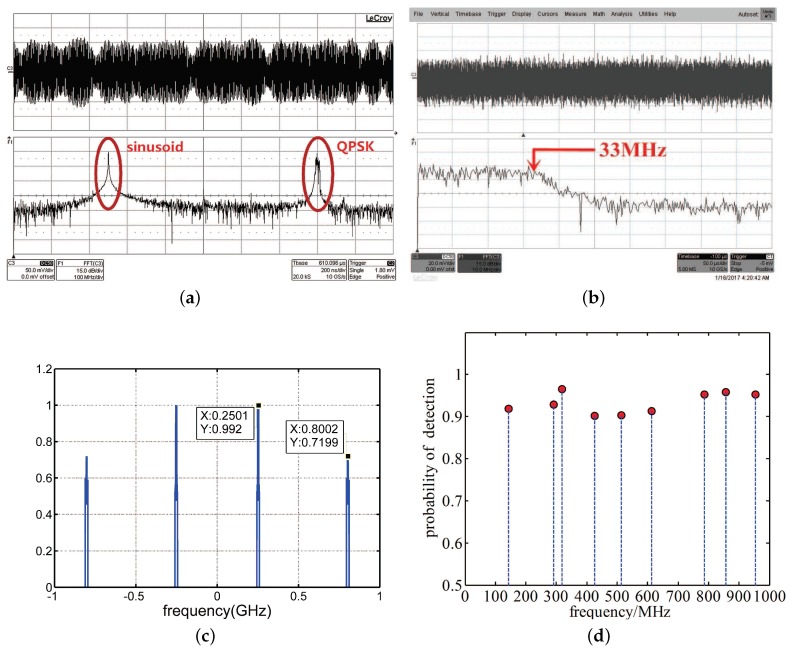
(**a**) The input signal and (**b**) Samples after LPF. (**c**) Detected power spectrum and (**d**) probability of detection.

To test the actual performance of the SCMWC prototype, we conducted a series of experiments. At first, we use the Keysight M8190A Arbitrary Wave Generator and Agilent N9936A to produce a signal consists of two discrete frequency bands: a pure sine waveform at 250 MHz and a quadrature phase shift keying (QPSK) signal at 800 MHz with 5 MHz bandwidth. And both amplitude is around −10dBm. Figure 16a,b depicts the time-domain and spectrum of both input signal and low rate samples. In Figure 16c, the detected power spectrum is presented. From the three figures above, we can see the accuracy of detected power spectrum is pretty high, which indicates our system can sense two bandlimited signals at 250 MHz and 800 MHz precisely.

Then, to test the stability, we conducted the following experiments. First using Matlab to generate nine carrier frequencies randomly in every 100 MHz. The frequency support is $F\;=\;\left[143,291,318,426,514,613,786,857,954\right]$ (MHz). Then, we randomly choose two frequencies among F to carry out same experiment as above. The total number of times running the experiments is 500. Correct detection is defined as ${\hat{S}}_{x}⊇S_{x}$ similarly. The result is shown in Figure 16d. As can be seen, during these experiments, the detection accuracy is pretty stable around 90% percent. This demonstrates the possibilities of the proposed SCMWC prototype to be used in actual CR system.

### 5.4. Comparisons with Related Work

In this section, we make some comparisons with other compressed wideband spectrum sensing hardware, mainly focusing on the performance and actual hardware complexity. As for performance, we compare with the first reported practical hardware that performs sub-Nyquist sampling based on MWC in [20]. From Table 2, we can see the most significant difference is compression ratio, that means we make it possible to use one low rate ADC to detect the whole sensing bandwidth. Also, the signal bandwidth is a little lower than that of MWC, which means the estimation accuracy improves a little to some extent.

Another comparison is concerned about hardware complexity in actual design. As shown in Table 3, SCMWC has a big advantage over MWC. In detail, SCMWC only needs one frond-end and most importantly, in designing the periodic sequences generator, we introduced to use GTX, which can highly reduce the complexity and size of the hardware. However, in MWC, two boards is used. One is called digital board aiming to produce sequences with a lot of shift registers (SR) on it. Another one is called analog board with the purpose of power allocation, mixing, low-pass-filtering and so on. It is apparent that SCMWC based spectrum sensing hardware has a simpler structure which makes it more suit for CR systems.

## 6. Conclusions

In this paper, we presented a power spectrum sensing scheme for CR system based on single channel modulated wideband converter and implemented it on circuit board. For the problem of hardware realization, we use commercial devices and each part of our circuit design is discussed in detail. The creative using of GTX to generate periodic sequences is flexible for designing and configuring process. Through software simulations and hardware experiments, we proved the feasibility of our prototype in wideband spectrum sensing for WSS signals. The proposed scheme can reduce the number of MWC’s physical parallel channels to one. Most importantly, the total sampling rate as well as hardware complexity is highly reduced. For future work, we consider to design a more practical device based on SCMWC for wideband spectrum sensing. Meanwhile, in order to detect a large number of signals, a more effective strategy with highly integrated circuit board should also be investigated.

## Figures and Tables

**Table 1:** Prototype specifications.

Parameter	Choice
signal model	N=4, B=15 MHz, $f_{nyq}=2$ GHz
number of period *m*	16
alteration rate	2 GHz
sign pattern length *M*	127
period $f_{p}$	2/127=15.7 MHz
filter cut-pff	33 MHz
sampling rate $f_{s}$	70 MHz

**Table 2:** Performance Comparisons between MWC and SCMWC.

Parameter	MWC	SCMWC
sensing bandwidth	2 GHz	2 GHz
maximum signal bandwidth	19 MHz	15 MHz
equivalent sampling rate	280 MHz	70 MHz (minimum is 15.7 MHz)
compression ratio	14%	3.5% (minimum is 0.78%)
daynamic range	49 dB	60.95 dB

**Table 3:** Complexity Comparisons between MWC and SCMWC.

Device	MWC	SCMWC
front-end	four sets	one set
periodic sequences generator	a lot of SR chips	one GTX
number of ADCs	4	1
synchronization requirement	Yes	No
size	two boards	one board

## References

[1] Haykin S. (2005). Cognitive radio: Brain-empowered wireless communications. IEEE J. Sel. Areas Commun..

[2] Cabric D., Mishra S.M., Brodersen R.W. Implementation issues in spectrum sensing for cognitive radios. Proceedings of the Conference Record of the Thirty-Eighth Asilomar Conference on Signals, Systems and Computers.

[3] Yucek T., Arslan H. (2009). A survey of spectrum sensing algorithms for cognitive radio applications. IEEE Commun. Surv. Tutor..

[4] Cabric D., Tkachenko A., Brodersen R.W. Spectrum Sensing Measurements of Pilot, Energy, and Collaborative Detection. Proceedings of the MILCOM 2006—2006 IEEE Military Communications Conference.

[5] Ariananda D.D., Leus G. (2012). Compressive Wideband Power Spectrum Estimation. IEEE Trans. Signal Process..

[6] Landau H.J. (1967). Necessary density conditions for sampling and interpolation of certain entire functions. Acta Math..

[7] Candes E.J., Romberg J., Tao T. (2006). Robust uncertainty principles: Exact signal reconstruction from highly incomplete frequency information. IEEE Trans. Inf. Theory.

[8] Donoho D.L. (2006). Compressed sensing. IEEE Trans. Inf. Theory.

[9] Mishali M., Eldar Y.C. (2011). Sub-Nyquist Sampling. IEEE Signal Process. Mag..

[10] Kirolos S., Laska J., Wakin M., Duarte M., Baron D., Ragheb T., Massoud Y., Baraniuk R. Analog-to-Information Conversion via Random Demodulation. Proceedings of the 2006 IEEE Dallas/CAS Workshop on Design, Applications, Integration and Software.

[11] Mishali M., Eldar Y.C. (2010). From Theory to Practice: Sub-Nyquist Sampling of Sparse Wideband Analog Signals. IEEE. J. Sel. Top. Signal Process..

[12] Herley C., Wong P.W. (1999). Minimum rate sampling and reconstruction of signals with arbitrary frequency support. IEEE Trans. Inf. Theory.

[13] Xu Z., Li Z., Li J. (2016). Broadband Cooperative Spectrum Sensing Based on Distributed Modulated Wideband Converter. Sensors.

[14] Cohen D., Eldar Y.C. (2013). Sub-Nyquist Sampling for Power Spectrum Sensing in Cognitive Radios: A Unified Approach. IEEE Trans. Signal Process..

[15] Sun W., Huang Z., Wang F., Wang X., Xie S. (2016). Wideband Power Spectrum Sensing and Reconstruction Based on Single Channel Sub-Nyquist Sampling. IEICE Trans. Fundam..

[16] Chiang R.I.C., Rowe G.B., Sowerby K.W. A Quantitative Analysis of Spectral Occupancy Measurements for Cognitive Radio. Proceedings of the IEEE Vehicular Technology Conference.

[17] Mishali M., Eldar Y.C. (2008). Reduce and Boost: Recovering Arbitrary Sets of Jointly Sparse Vectors. IEEE Trans. Signal Process..

[18] Chen S., Donoho D. Basis pursuit. Proceedings of the 1994 28th Asilomar Conference on Signals, Systems and Computers.

[19] Mallat S.G., Zhang Z. (1993). Matching pursuits with time-frequency dictionaries. IEEE Trans. Signal Process..

[20] Mishali M., Eldar Y.C., Dounaevsky O., Shoshan E. (2011). Xampling: Analog to digital at sub-Nyquist rates. IET Circuits Devices Syst..

[21] ADL5801. http://www.analog.com/media/en/technical-documentation/evaluation-documentation/ADL5801.pdf.

[22] 7 Series FPGAs GTX/GTH Transceivers. http://www.xilinx.com/support/documentation/user_guides/ug476_7Series_Transceivers.pdf.

[23] Virtex-5 FPGA RocketIO GTX Transceiver User Guide. https://china.xilinx.com/support/documentation/user_guides/ug198.pdf.

[24] Israeli E., Tsiper S., Cohen D., Shoshan E., Hilgendorf R., Reysenson A., Eldar Y.C. Hardware calibration of the modulated wideband converter. Proceedings of the 2014 IEEE Global Communications Conference.

[25] Chen L., Jin J., Gu Y. A calibration system and perturbation analysis for the Modulated Wideband Converter. Proceedings of the IEEE 10th International Conference on Signal Processing.

[26] Chen Y., Mishali M., Eldar Y.C., Hero A.O. Modulated wideband converter with non-ideal lowpass filters. Proceedings of the 2010 IEEE International Conference on Acoustics, Speech and Signal Processing.

[27] Liu Z.M., Huang Z.T., Zhou Y.Y. (2012). An Efficient Maximum Likelihood Method for Direction-of-Arrival Estimation via Sparse Bayesian Learning. IEEE Trans. Wirel. Commun..

[28] RF Cafe Website. http://www.rfcafe.com/references/electrical/ip3.htm.

